# Nondifferentiable activity in the brain

**DOI:** 10.1093/pnasnexus/pgae261

**Published:** 2024-07-01

**Authors:** Yasuhiro Tsubo, Shigeru Shinomoto

**Affiliations:** College of Information Science and Engineering, Ritsumeikan University, Osaka 567-8570, Japan; Research Organization of Open Innovation and Collaboration, Ritsumeikan University, Osaka 567-8570, Japan; Graduate School of Biostudies, Kyoto University, Kyoto 606-8501, Japan

**Keywords:** spike trains, cross-correlograms, monosynaptic connectivity, nondifferentiable fluctuations

## Abstract

Spike raster plots of numerous neurons show vertical stripes, indicating that neurons exhibit synchronous activity in the brain. We seek to determine whether these coherent dynamics are caused by smooth brainwave activity or by something else. By analyzing biological data, we find that their cross-correlograms exhibit not only slow undulation but also a cusp at the origin, in addition to possible signs of monosynaptic connectivity. Here we show that undulation emerges if neurons are subject to smooth brainwave oscillations while a cusp results from nondifferentiable fluctuations. While modern analysis methods have achieved good connectivity estimation by adapting the models to slow undulation, they still make false inferences due to the cusp. We devise a new analysis method that may solve both problems. We also demonstrate that oscillations and nondifferentiable fluctuations may emerge in simulations of large-scale neural networks.

**Significance Statement**

Modern technology allows us to record a large number of neuronal spike trains in parallel, providing information about inter-neuronal connectivity and background activity. Here, we show that cross-correlations exhibit not only slow undulations but also a differentially discontinuous cusp at the origin, even if neurons are not connected. We developed a new analysis method to remove their negative influence on connectivity estimation and show that a cusp in the cross-correlation is a sign of highly irregular or nondifferentiable changes in neuronal activity. Our approach opens new ways to analyze inter-neuronal connections and to understand the dynamic mechanisms underlying neural information processing in the brain.

## Introduction

Technological advancements have made it possible to record signals from multiple neurons for hours or days (1–5). The number of recorded neurons has doubled every seven years as with Moore’s law for semiconductor integrated circuits (6). This number now exceeds several thousand even for detecting electrical signals with submillisecond resolution (7–9). This unprecedented increase in the amount of data is expected to change our fundamental understanding of neuronal functions in the brain, just as semiconductor technologies have changed our daily life.

There have been various proposals to use multiple spike signals, particularly for analyzing inter-neuronal connections (10–16). It all started more than a half-century ago when Perkel, Gerstein, and Moore proposed to estimate the monosynaptic connectivity by capturing the interdependence between neuronal firings. This was done by detecting the significant deviations from a uniform distribution in a cross-correlation histogram a.k.a. a cross-correlogram (CC) of spike trains recorded from a pair of neurons (17). Nevertheless, the classical analysis methods make many false inferences because *in vivo* neuronal firings are originally correlated due to background activity even if a pair of neurons are not monosynaptically connected. There have been many attempts to improve the estimation (18–29), most of which involved the adaptation of models to variations that appeared in the CCs *ex post facto* by regarding them as unwelcome disturbers, but without determining the underlying causes.

Neuroscientists have seen many CCs exhibiting a sharp peak at the origin and presumed that such a peak may have been caused by common inputs from locally connected neurons. Here we analyze a large number of biological CCs and explore their possible causes. With mathematical considerations, we conclude that a CC can exhibit both smooth undulation and a sharp peak (or nonsmooth cusp) even if neurons are not monosynaptically connected. Namely, a CC exhibits smooth undulation if a pair of neurons are subjected to smooth oscillations, while it exhibits a cusp if there are highly irregular fluctuations that are not mean-square differentiable (30), which we call “nondifferentiable.”

While modern analysis methods have achieved a reasonable estimation of monosynaptic connectivity by adapting the models to slow undulation, they still make false inferences when a CC exhibits a sharp cusp at the origin. Accordingly, we revise the analysis method GLMCC developed by one of the authors and others (28) into a new method “ShinGLMCC” by allowing the model to adapt to not only undulation but also a cusp.

To test the reliability of analysis methods, we perform simulations of a large-scale network of neurons interacting with fixed synaptic connectivity. We find that the network may exhibit nondifferentiable activity similar to what we have observed in biological spike trains. Theoreticians have known that coherent synchronous activity may emerge not only in neural networks (31–38) but also in various self-excitation processes (39). It is noteworthy that pioneering neural network modeling studies have shown not only oscillation but also a cusp in the spike correlation function (40–46). While they have discussed possible functions for coherent dynamics in neuronal information processing, they have not paid much attention to the sharp temporal profile of the emergent fluctuating activity. This might be partly due to the lack of a sufficient amount of experimental data at that time.

We also devise fast algorithms for both GLMCC and ShinGLMCC and provide a user-friendly software package so that researchers can obtain a neuronal connection diagram and individual analysis details for all neuron pairs from a large number of recorded spike trains.

## Results

### Cross-correlograms of biological data

**Fig. 1.**
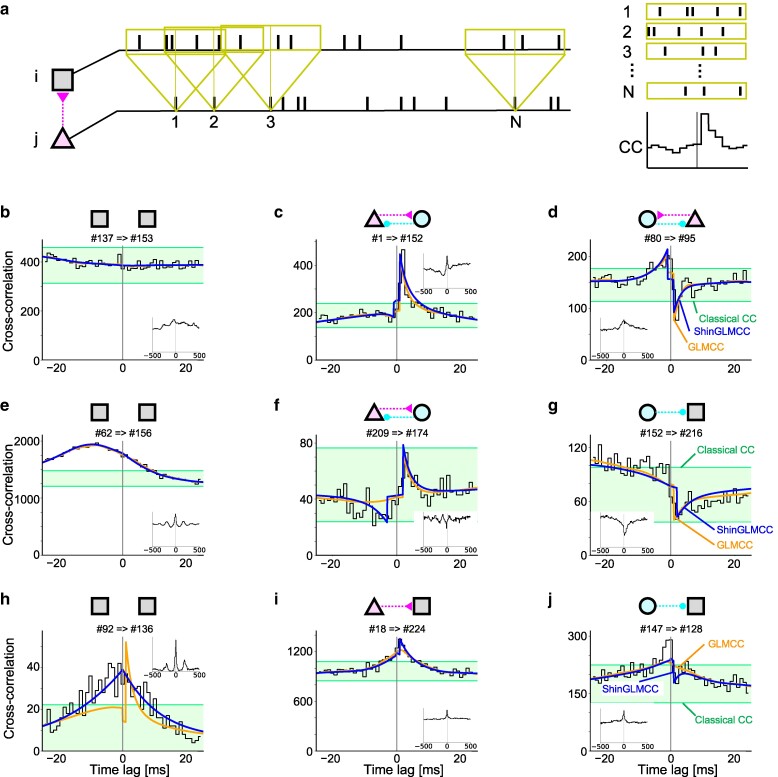
A variety of CCs obtained from biological spike trains. a) Process of constructing a CC from a pair of spike trains. b) A flat CC. c and d) A hump and a dip at several milliseconds right side from the origin. e) A CC exhibits a large undulation. f and g) A hump and a dip immersed in large undulating variations. h) A CC exhibits a cusp at the origin. i and j) A hump and a dip immersed in a cusp. Shaded belts represent a confidence interval suggested by the Classical CC analysis. Orange and blue lines represent inferences of GLMCC and ShinGLMCC, respectively. Schematic diagrams in the above represent putative connections determined by ShinGLMCC. Triangles and circles represent putative excitatory and inhibitory neurons, while squares are for those with undetermined characteristics.

The CC is a basic device to display the potential interdependence of two units. This can be constructed by simply superposing the spike times of one train measured relative to every spike of another train (Fig. 1a). Here we demonstrate a variety of CCs obtained from publicly sourced data of spike trains recorded in parallel from wide brain regions of a freely moving rat using a silicon probe called Neuropixels (2, 47).

#### Flat distribution in CCs

If a pair of neurons has generated spikes independently of each other, their CC is expected to be flat apart from statistical fluctuations that occur when counting spikes in each bin. A large fraction of neuron pairs in the cortex display flat CCs, implying that synaptic connections between them may be either absent or insignificant, as shown in Fig. 1b.

If synaptic connections exist between neurons, they will induce statistical interdependence between two spike trains, and their CC is expected to display either a hump for excitatory monosynaptic connectivity (Fig. 1c) or a dip for inhibitory connectivity (Fig. 1d) several milliseconds from the origin. A “Classical CC analysis” seeks to detect a significant deviation from the flat distribution based on the stochastic point process theory (17, 48). The confidence intervals representing natural statistical fluctuations are depicted as shaded belts in Fig. 1.

#### Smooth undulation in CCs

While Classical CC analysis gives plausible inferences for typical cases (Figs. 1b–d), it does fail in many cases, particularly when there are large undulating variations that exceed the assigned confidence interval as in Figs. 1e–g. There have been many efforts to reduce the influence of such large variations (18–27). Most of them attempted to smooth out such large variations by regarding them as unwelcome disturbers. For instance, one of the authors and others developed a method called GLMCC, in which a generalized linear model adapts a slowly fluctuating function to existing variations in the CC, and accordingly, reduces false inferences (28). The analysis method was further revised by introducing the likelihood ratio test into the determination of connections (29). The slowly fluctuating functions and monosynaptic impacts determined by GLMCC are depicted in Fig. 1. Large undulations are well represented by GLMCC, particularly in Figs. 1e–g.

#### A cusp in CCs

By analyzing a large number of biological spike trains, however, we have newly found other difficult cases that even the GLMCC makes dubious inferences for. This occurs particularly when a CC exhibits a seemingly nonsmooth cusp at the origin as represented in Figs. 1h–j. In these cases, GLMCC does not seem successful in representing large variations in individual CCs.

In the present study, we develop ShinGLMCC from GLMCC so that the adaptation function can bend at the origin as shown in Figs. 1h–j. Before developing the analysis method, we consider the possible cause for the cusp that appears in CCs in the following section.

### Models for the variations in CCs

To tackle the problem that accompanies both the undulation and the cusp in CCs, we begin by exploring their possible causes using mathematical models for spike generation (Materials and Methods).

#### Producing smooth undulations in a CC

**Fig. 2.**
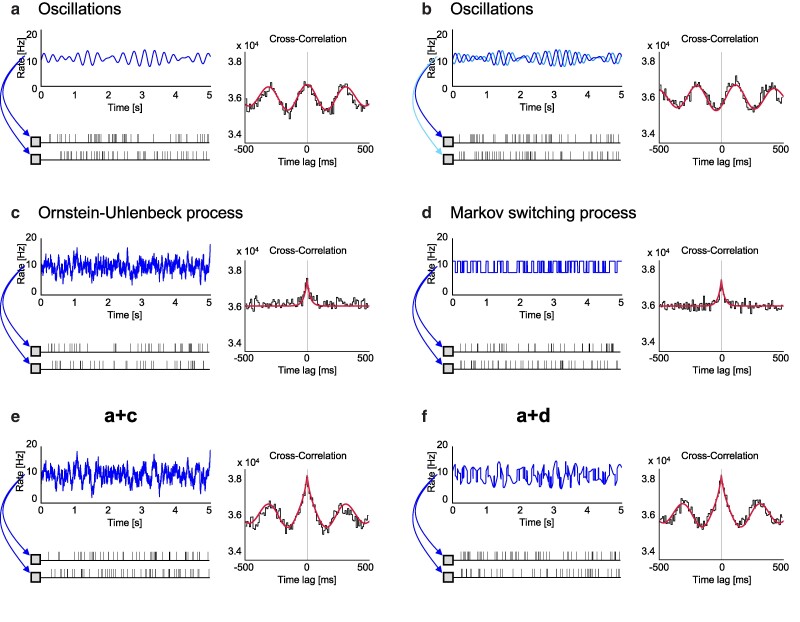
Synthetic spike trains producing the smooth undulation and a cusp in CCs. a) Neurons are commonly modulated with smooth oscillations. b) The case of smooth modulation with a time lag. c) Neurons are commonly modulated with rates that are nondifferentiable everywhere, according to the Ornstein–Uhlenbeck process. d) Neurons whose firing rates switch between high and low values randomly in time, according to the Markov switching process. e) Neurons are modulated by oscillations and nondifferentiable fluctuations (a and c). f) Neurons are switching between high and low oscillatory states (a and d). Red lines are analytically obtained CCs for individual models.

Before addressing the aspect of the cusp, let us first consider the appearance of smooth undulation in a CC. Smooth undulation may be produced by adding smooth oscillations in the firing rates of a pair of neurons, mimicking the situations that biological neurons are influenced by (smooth) brainwave oscillations (Materials and Methods). The basic frequency of the undulation in a CC is identical to the frequency of the rate modulation. The undulation may have a peak at the CC’s origin if two neurons are influenced in phase. The undulation may exhibit damping if the frequency components of the rate modulation are distributed (Fig. 2a). The peak of the undulation in a CC may be shifted if a pair of neurons receive oscillatory modulation with a certain time lag (Fig. 2b). CCs constructed from a given data set exhibit fluctuations in counting spikes as long as the spike trains are of finite length. Nevertheless, such counting fluctuations can be minimized if spike trains are sufficiently long because the number of spike counts in each bin of the CC histogram increases with the length of spike trains (Supplementary Material A).

#### Producing a cusp in a CC

While a CC can show smooth undulation due to background oscillations as described above, it will never show a (nonsmooth) cusp as long as external influences are smooth. A necessary and sufficient condition for having a smooth correlation function is that the original rate processes are “mean-square differentiable” (30). Accordingly, a pair of neurons that show a cusp in the CC must have been modulated by nondifferentiable activity.

We have explored possible spike-generation processes such that the CC shows a cusp at the origin (Materials and Methods). One possible situation is that neurons generate spikes with time-dependent firing rates that are nondifferentiable everywhere, as exemplified by the “Ornstein–Uhlenbeck process” (Fig. 2c) (49–53).

Note that this model process assumes that two neurons are not monosynaptically connected, because spikes of one neuron have never influenced the spike generation of another neuron. Nevertheless, their firings are not independent, because they are influenced by identical background fluctuations. The model simulation mimics the situation that neurons are influenced by common fluctuations that are nondifferentiable everywhere.

We also found another situation in which the CC exhibits a cusp. Namely, two neurons generate spikes according to firing rates that switch between high and low values randomly according to the “Markov switching process” (Fig. 2d) (53–58). While the underlying rate is not nondifferentiable everywhere, it exhibits (more drastic) discontinuous changes at a finite rate. Two neurons are not monosynaptically connected either, because spikes of one neuron do not influence the firing of another neuron.

Different rate processes such as the Ornstein–Uhlenbeck process and Markov switching process may end up with analytically identical CCs exhibiting a cusp at the origin. We cannot identify a time profile of the original rate process accurately from the recorded spike trains because spikes are a mere random realization of a stochastic process. Nevertheless, we can obtain a sharp profile of the CC from sufficiently long spike sequences because the number of spikes in histogram bins can be increased with the recording duration, such that the relative influence of the spike count fluctuations can be made sufficiently small. The cusp at the origin of a CC may remain even sharply if we can collect longer time series (Supplementary Material A).

Figures 2e and f depict examples of a pair of spike trains derived from the rate process in which smooth oscillation of Fig. 2a and nondifferentiable fluctuations of Figs. 2c and d are mixed. In this case, the CC exhibits not only smooth undulation but also a cusp at the origin, as seen in the biological data in Figs. 1h–j.

### Population activity of biological data

**Fig. 3.**
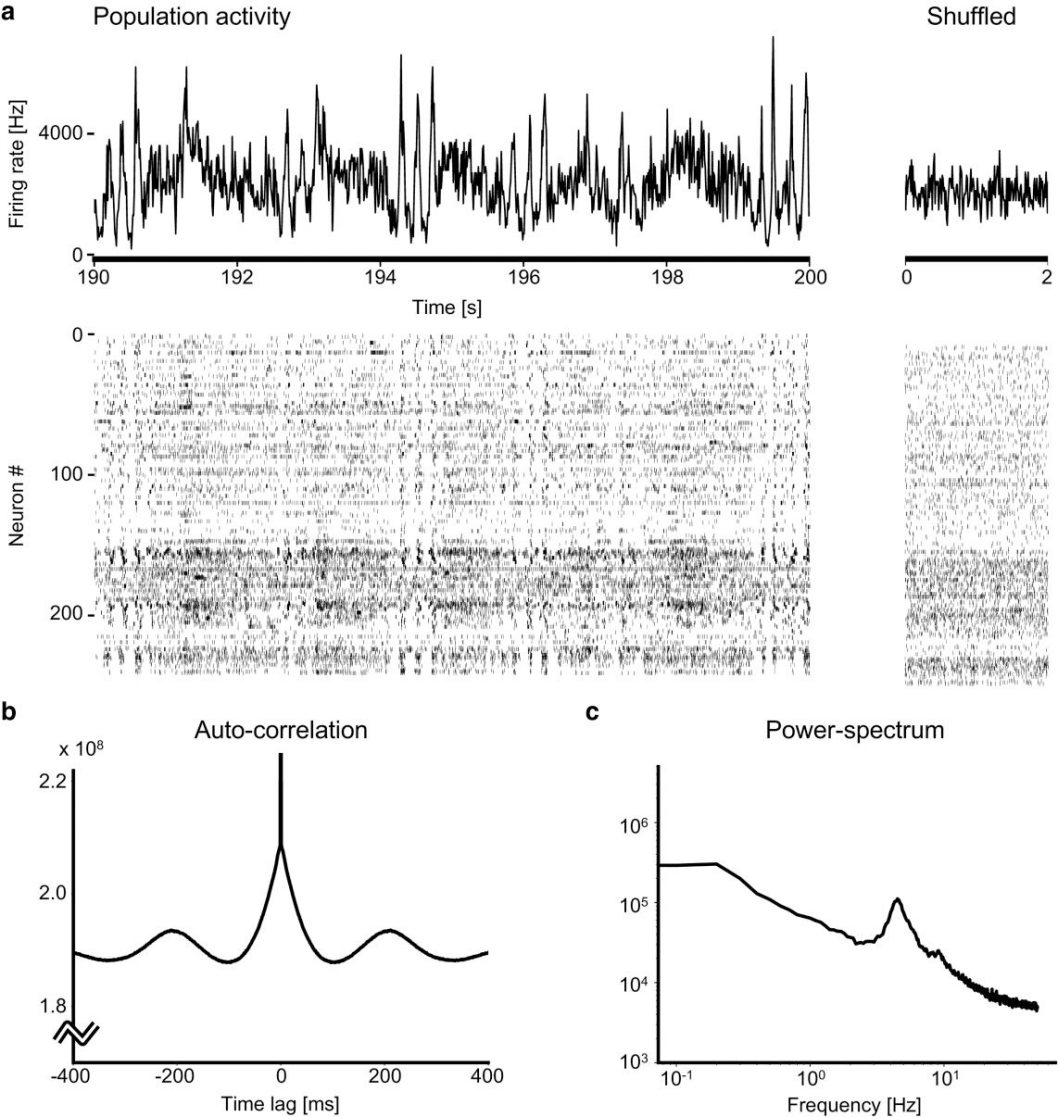
Nondifferentiable fluctuations in the population activity of biological neurons. a) (Left-top) A time histogram of the number of spikes of 242 units for 10 s, with a time bin of 10 ms. (Left-bottom) Spike raster plots of 242 units. (Right-top) A time histogram of a shuffled spike train for 2 s. (Right-bottom) Raster plots of shuffled spike trains. b) An autocorrelation histogram of the summed spike train (1 ms bin). c) A power spectrum of the summed spike train.

To observe the nondifferentiable feature of the background activity, which may have caused a cusp at the origin of pairwise CCs, we summed a large number of spike trains into a single spike train, expecting the background activity to appear in this single spike train’s firing rate. A time histogram of an entire spike train of 242 units recorded from the visual cortex, hippocampus, and thalamus is depicted in Fig. 3a. Slow oscillations and seemingly nondifferentiable activity coexist in an entire firing rate. In the raster plots of spike trains, we observe a striped pattern, indicating that a subset of neurons exhibits seemingly sharp fluctuations in their firing rates. To see the degree of temporal variability of the original data, we show shuffled spike trains on the right side of Figure 3a as a reference.

The autocorrelation of the summed spike train exhibits both slow undulation and a cusp, in addition to the delta peak of self-spike counts (Fig. 3b). This suggests that there is a sharp coherent activity in the brain. To test whether the central peak of the autocorrelation is a nonsmooth cusp or it is just a smooth hump, we applied jittering or dithering so that spike times are randomly shifted around the original times (21, 59, 60) and found that the peak of the autocorrelation histogram collapsed into a smooth hump, even with dithering spike times by a few ms (Supplementary Material B). Furthermore, we compared a piecewise differentially discontinuous function with a differentially continuous function for their goodness of fit to the autocorrelation histogram of the summed spike train, with the delta peak that is due to self-spike counts is removed. As a result, the central peak of the autocorrelation histogram is better represented as a cusp given by a piecewise differentially discontinuous function than as a smooth peak given by a differentially continuous function, thus supporting the presence of nondifferentiable activity in the brain (Supplementary Material C).

Figure 3c shows the power spectrum of an entire spike train. It exhibits a hump with a frequency close to 5 Hz, which may represent the theta rhythm oscillation that is present near the hippocampus. This may have led to the smooth undulation in the autocorrelation. We also analyzed other data of Ref. (2, 47) recorded from the frontal cortex including motor areas and the posterior cortex including visual areas, in which cases the oscillation is less pronounced (Supplementary Material D). Interestingly, a nonsmooth cusp was observed in their autocorrelation histograms, even though oscillations were not noticeable.

### ShinGLMCC

In the preceding section, we showed that biological data exhibit not only slow undulation but also a cusp at the origin of the CCs. While recent methods for estimating synaptic connections, e.g. GLMCC, have overcome the difficulty of undulation by fitting a smooth function to a given CC (Figs. 1e–g), they do not seem to have successfully captured the cusp in the CC (Figs. 1h–j). This may be because they assumed even smoothness over the time axis of the CC. To further improve the estimate, we need to remove the influence of the cusp.

Here we free the adaptation function from the smoothness at the origin of the time axis. This can be done by removing the penalty at the origin in the prior distribution of the generalized linear model. We call this revised model ShinGLMCC (Materials and Methods).

Although ShinGLMCC has an adaptation function that resembles GLMCC’s, they differ particularly near the origin, whose detail is crucial in determining monosynaptic connections. The adaptation function and synaptic impacts determined by ShinGLMCC are depicted in Figs. 1b–j. They seem successful in representing not only undulation but also a cusp of the variation in CCs.

### Comparison of analysis methods on biological data

We applied three analysis methods (Classical CC, GLMCC, and ShinGLMCC) to all pairs of spike trains of 242 neurons recorded from the brain of a rat and obtained connection matrices for all directed links (242×(242−1)=58,322).

**Fig. 4.**
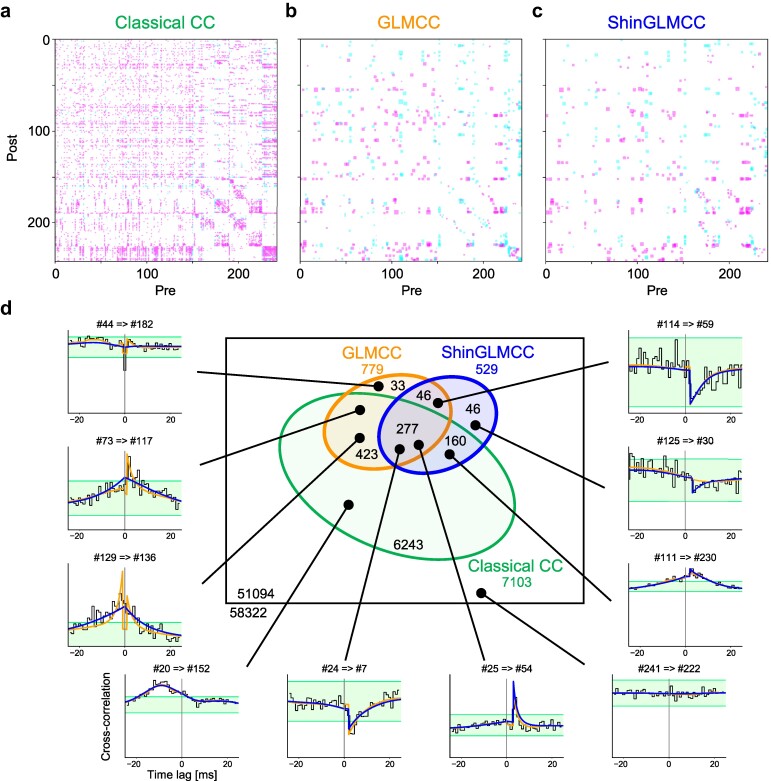
Connections estimated for biological data. a)–c) Connection matrices obtained for biological data using the Classical CC method, the GLMCC method, and the newly developed ShinGLMCC method, respectively. Magenta and cyan squares in each matrix represent excitatory and inhibitory connections, respectively estimated by each analysis method. d) Venn diagram representing the relationships between determined connectivities. Sample CCs are displayed along the periphery.

The Classical CC analysis has suggested 7,103 connections, occupying 12% of all possible links (Fig. 4a). As discussed in the previous section, Classical CC analysis tends to suggest many spurious connections due to large variations in CCs that are found in the biological data.

The GLMCC has suggested 779 connections, occupying 1.3% of possible links (Fig. 4b), obviously improving the inference from Classical CC analysis, by eliminating many seemingly spurious connections.

ShinGLMCC, our new method, suggested 529 connections, occupying 0.9% of possible links (Fig. 4c). A large fraction (61%) of connections suggested by ShinGLMCC are consistent with 41% of connections suggested by GLMCC (Fig. 4d).

The cases for which ShinGLMCC and GLMCC make different suggestions are displayed in Figs. 1h–j and in the periphery of a Venn diagram of Fig. 4d. As has been discussed in the previous sections, ShinGLMCC appears to provide more reliable inferences than GLMCC, particularly for the CCs that exhibit a cusp.

The cusp CC indicates that the local activity is nondifferentiable. To see the difference in the local dynamics of neuron pairs, we have compared ShinGLMCC and GLMCC for their goodness of fit to each CC. In the Supplementary Material E, we show matrices indicating the neuron pairs for which ShinGLMCC fits the CC better (the likelihood is higher) than GLMCC. There are significant differences in the cusp CC dominance between different brain regions.

Furthermore, we examined the extent to which the excitatory or inhibitory characteristics of identified connections are consistent for individual sending neurons, in terms of the excitatory–inhibitory (E–I) dominance index proposed in Ref. (28), which is defined as $d_{\mathrm{ei}}=(n_{e}-n_{i})/(n_{e}+n_{i})$, where $n_{e}$ and $n_{i}$ are the numbers of identified excitatory and inhibitory connections projecting from each neuron, respectively. We have shown the distributions of the E–I dominance index for Classical CC, GLMCC, and ShinGLMCC in Supplementary Material F. The Classical CC, GLMCC, and ShinGLMCC gave connections to 241, 189, and 142 neurons out of a total of 242 neurons. The average absolute values of E–I index $\left|d_{\mathrm{ei}}\right|$ were 0.78, 0.81, and 0.86, and the fractions of neurons expressing perfect consistency ($d_{\mathrm{ei}}=1$ or −1) were 0.28, 0.69, and 0.75, respectively, suggesting that ShinGLMCC may have provided a reliable inference.

### Simulations of a large-scale network of neurons

While inter-neuronal connections determined by the new algorithm ShinGLMCC seem reasonable in the apparent shapes of individual CCs, we cannot be certain of the decision from the available biological data. Here, we compare the performance of Classical CC, GLMCC, and ShinGLMCC in estimating connectivity using synthetic data from a large-scale network of neurons interacting through given synaptic connections.

We performed simulations of networks of 1,000 (800 excitatory and 200 inhibitory) model spiking neurons (Materials and Methods). The mathematical model is similar to what has been used in Ref. (29), but we have explored wider parameter ranges by increasing the strength of interneuronal connectivity such that the system exhibits nonstationary activity.

#### Emergence of nondifferentiable activity in large-scale network simulations

**Fig. 5.**
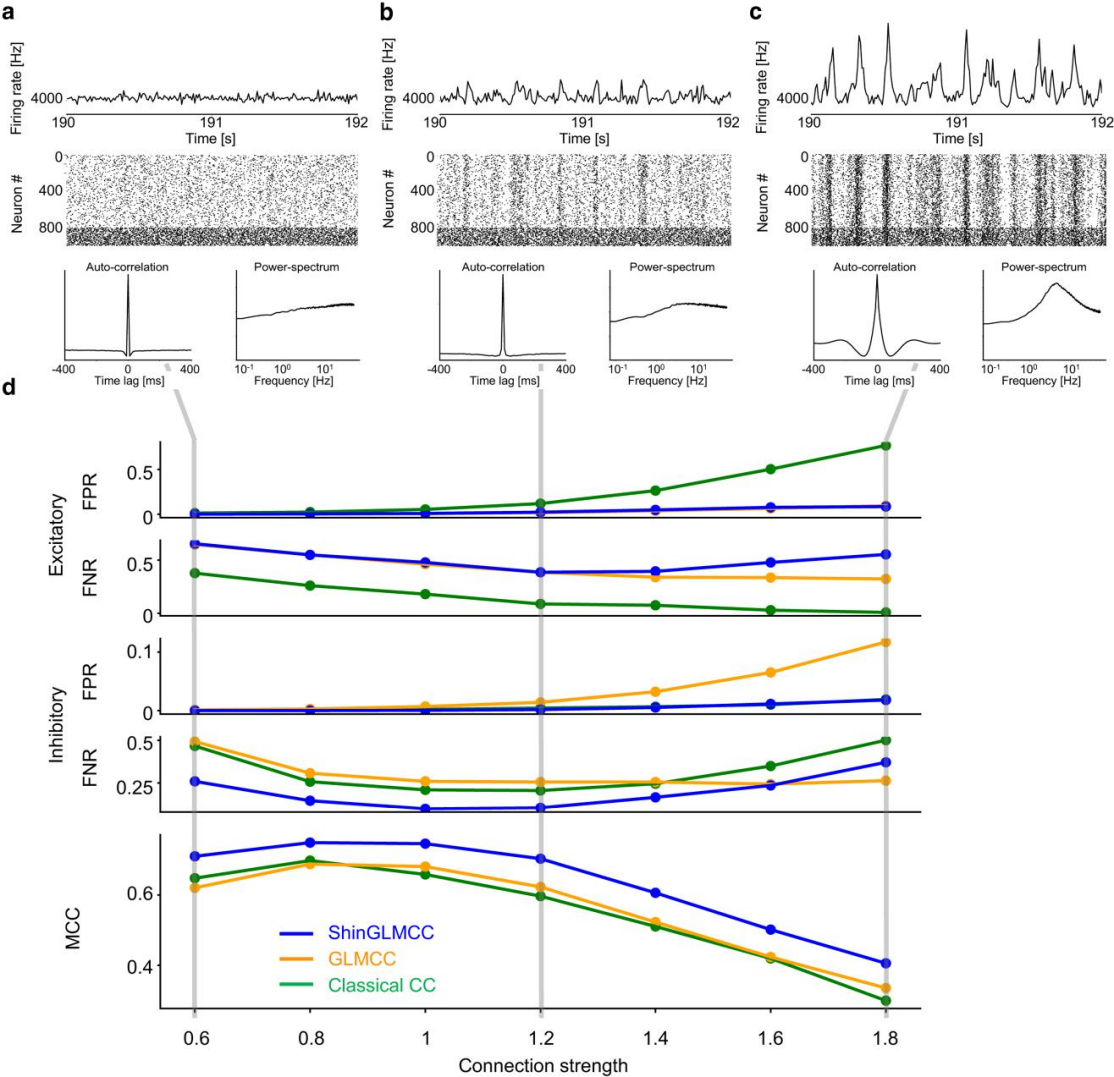
Population activity of a network of 1,000 spiking neuron models. a–c) Time histograms of the number of spikes of a total population of neurons, their spike raster plots for a time window of 2 s, autocorrelation functions, and power spectrum of summed spike trains for the simulations of a network of average connection strength A=0.6,1.2, and 1.8. d) Estimation performances of Classical CC, GLMCC, and ShinGLMCC, as represented by the false positive rate (FPR) and false negative rate (FNR) for excitatory and inhibitory categories computed for 100 neurons (smaller the better), and the Matthews correlation coefficient (MCC) from FPs and FNs (larger the better), plotted against *A*.

With a relatively weak connection strength, the entire system exhibits a stationary time series in which neurons fire asynchronously (Fig. 5a). By increasing the strength of synaptic connections while maintaining a given connectivity matrix, an entire neural network starts to exhibit synchronous burst firings intermittently, displaying irregular stripes in raster plots (Fig. 5b). In this strong connectivity regime, a time histogram and raster plots resemble those of real biological neurons shown in Figure 3a. By increasing the strength of the connections further, the system starts to exhibit oscillation, in addition to the irregular burst firings (Fig. 5c). The autocorrelation exhibits a cusp and oscillation similar to the biological data as shown in Fig. 3b. This implies that both the smooth oscillation and the nondifferentiable fluctuations may emerge even in numerical simulations of a large-scale network of neurons in a regime of strong connectivity.

#### Evaluation of various analysis methods

We evaluate the ability of Classical CC, GLMCC, and ShinGLMCC to infer connectivity by applying them to 100 spike trains selected from the entire set of 1,000 neurons. We counted the numbers of false positives (FPs) or spurious connections and false negatives (FNs) or missing connections for excitatory and inhibitory categories, and the Matthews correlation coefficient (MCC) evaluated the total performance (Materials and Methods). Fig. 5d shows how the FPR and FNR for excitatory and inhibitory categories as well as MCC change with the average strength of inter-neuronal connectivity.

The Classical CC analysis registered a large number of FPs for excitatory connections (Fig. 5d, first row), as seen in the connection matrix computed for the experimental data (Fig. 4a), particularly when the connection strength is increased and the raster plots exhibit stripes. While GLMCC and ShinGLMCC have shown smaller excitatory FPR, their excitatory FNR turned out to be larger than that of Classical CC (Fig. 5d, second row). This may be because we have set their detection criteria very strict (significance level $\alpha =10^{-4}$). As a result, Classical CC is competing with GLMCC by the criterion of the total score MCC (Fig. 5d, fifth row), in which four scores (FPR and FNR for excitatory and inhibitory categories) are treated equally. Even in this criterion, ShinGLMCC is superior to GLMCC as well as Classical CC, particularly registering fewer FPs for the inhibitory category (Fig. 5d, forth row).

## Discussion

In this study, we have found that CCs of biological spike trains exhibit not only slow undulation but also a cusp at the origin, in addition to possible signs of monosynaptic connectivity. We explored possible mechanisms and found that such undulation and a cusp may have been caused by smooth oscillations and nondifferentiable latent activity in the background, respectively. We developed a new method for estimating connectivity called ShinGLMCC that successfully eliminates the influence of a cusp. We have also performed a simulation of a large-scale network of spiking neuron models and found that the model network may also exhibit synchronous burst firings, inducing nondifferentiable fluctuations similar to what we have observed in real biological data.

While the concept of nondifferentiability cannot be rigorously proven with data of finite recording duration, the biological data analyzed here are consistent with such a hypothesis. Namely, we have found CCs that express a cusp as shown in Figs. 1h–j, and the autocorrelation of a summed spike train exhibited a cusp as in Fig. 3b. Therefore, we may conclude that the latent dynamics in the brain may have been sharp enough to be described as “nondifferentiable” at least on the timescale of a few ms. By focusing our attention on spontaneous activity, we may realize that we have often seen sharp striped patterns in spike raster plots of biological data, as shown in Fig. 3a. It is noteworthy that Riehle et al. have reported not only the presence of sharp synchrony between different neurons but also their context-dependent switching in the monkey brain (61). Accordingly, the concept of nondifferentiability may apply to a wide range of biological data.

The ShinGLMCC as well as the GLMCC automatically adapt a function to a CC of a given pair of neurons, independently of other pairs. As the fitting function of ShinGLMCC may represent the background activity properly, there is a possibility that neuron pairs that have similar fitting functions might be sharing common inputs. It may be interesting to use such information when analyzing the overall network structure of recorded neurons.

We also have found that synchronous firings occur in numerical simulations displaying stripes in raster plots (Figs. 5b–c) and suggested that these may be related to the nondifferentiable activity we found in real brains. Note that a symmetrical peak or a cusp may emerge at the origin of CCs even if there is no nontrivial local connection structure because model neurons are uniformly randomly coupled in a network. It would also be interesting to study how emergent properties may further depend on the distribution of connection strengths or connection patterns. In terms of simulation, however, it should be noted that neural network models are very sensitive to the connection parameters and tend to be unstable (62). Here, we performed numerical simulations of networks with log-normally distributed excitatory connection strengths because this is a rare condition under which the neurons may exhibit irregular firing at low firing rates, consistent with experimental findings (63).

Theoreticians have known that spontaneous coherent dynamics may emerge in large-scale networks and have discussed their possible functions in information processing (31–39). Pioneering neural network modeling studies have shown not only oscillation but also a cusp in the spike correlation function (40–46). While they discussed the possible network structures that might have produced such spike correlations, it appears they did not pay much attention to the possibility of such a cusp being realized in biological networks. This is presumably because the amount of experimental data was not sufficient at that time.

Here we found that cross-correlations of biological spike trains exhibit a nonsmooth cusp at zero time lag, and examined the hypothesis of nondifferentiable latent dynamics in the brain. While there has not been much debate about whether the latent dynamics of the brain are continuous or discontinuous, or, smooth or nonsmooth, there have been some important suggestions for the possibility of discontinuous dynamics: Abeles et al. have suggested that cortical activity may switch between discrete states (54); Mochizuki and Shinomoto developed a method of detecting analog vs. digital codes in the brain (58); Stringer et al. suggested that the brain encodes a high-dimensional latent state (64). The current study may support such a discontinuity hypothesis on the basis of biological data, thus raising an issue for the dynamics of latent states in the brain.

We also have devised fast algorithms for the estimation methods GLMCC and ShinGLMCC and provided user-friendly software packages for researchers to obtain connection diagrams and individual analysis details for all neuron pairs from a given set of recorded spike trains. The algorithm of ShinGLMCC completes 58,322 pairs in about 10 minutes on a Mac Pro (2019, 28cores). While the new ShinGLMCC algorithm provides highly accurate estimates of monosynaptic connections, it would be worthwhile to look for further improvements, possibly using Bayesian methods, particularly when the number of spikes is small. It should be noted, however, that there is an essential limitation in that the connection cannot be inferred if the number of spikes is too small. For instance, even if we observe that a neuron fired several times immediately after another neuron fired, we cannot conclude that this is due to excitatory coupling because they may simply be coincidental. The number of spikes required for the statistical significance is given theoretically by Aertsen and Gerstein (48). Furthermore, Kobayashi et al. have given the limit more explicitly in terms of postsynaptic potentials (Equation (3) and Table 1 of Ref. (28)). For instance, to estimate EPSPs of the order of 1 mV for neurons firing at several Hz, we typically need a recording that is much longer than an hour.

**Table 1.** Parameters for neuron models

$\tau_{m}^{\mathrm{excitatory}}$ , $\tau_{m}^{\mathrm{inhibitory}}$ [ms]	[10, 20], [5, 10]
$V_{L}$ , $V_{E}$, $V_{I}$ [mV]	− 70, 0, −80
$\tau_{s,e}$ , $\tau_{s,i}$ [ms]	1, 2
$\tau_{1}$ , $\tau_{2}$ [ms]	10, 200
$\omega^{\mathrm{excitatory}}$ , $\omega^{\mathrm{inhibitory}}$ [mV]	− 56, −57
$\alpha_{1}^{\mathrm{excitatory}}$ , $\alpha_{2}^{\mathrm{excitatory}}$ [mV]	Gauss(10, 0.3), 1
$\alpha_{1}^{\mathrm{inhibitory}}$ , $\alpha_{2}^{\mathrm{inhibitory}}$ [mV]	10, 0.2

While the recorded neurons are still a mere small portion of the entire neuronal network, the disclosure ratio is rapidly increasing. The number of neuronal pairs that should be examined for potential connectivity increases much more rapidly with the order of the square of the number of neurons. With a suitable and fast analysis tool of this kind, we will be able to explore the difference in neuronal circuitry across different functional regions in the brain.

## Materials and methods

### Cross-correlogram

The CC is a means to display the potential interdependence of two units with a time delay *t*, defined as $c_{ij}(t)\equiv \bar{s_{i}(t^{\prime })s_{j}(t^{\prime }+t)}$, where $\bar{\cdots }$ represents an average over $t^{\prime }$, $s_{i}(t)$ and $s_{j}(t)$ are spike trains of *i*th and *j*th neurons, respectively (Fig. 1a). For a pair of independent spike trains, $c_{ij}(t)$ is generally flat, being accompanied by sample fluctuations (Fig. 1b). The classical theory instructed that $c_{ij}(t)$ may exhibit humps or dips at several milliseconds from the origin if two neurons are interacting via excitatory or inhibitory synapses, respectively (Figs. 1c and d), and devised a method of detecting them as an evidence of data outlying a given statistical significance level (17, 48).

However, CC may exhibit large fluctuations even if two neurons are not directly connected, because they may be receiving common background activity in the brain. We have seen that many CCs obtained from biological spike trains exhibit large undulation and a cusp (Figs. 1e–j). Hence, the Classical CC analysis often fails in its estimation.

### Model consideration of various variations in CCs

To determine possible mechanisms by which CCs exhibit undulation and a cusp, we ran inhomogeneous Poisson processes, such that spikes are derived from given instantaneous rates. Their rates should contain coherent parts so that the CC exhibits large deviations that exceed normal statistical fluctuations.

#### Models producing smooth undulation in a CC

The smooth undulation observed in a CC may be produced by simulating two neurons whose firing rates are modulated with smooth oscillations, as represented by $r_{1}(t)=r_{1}+a_{1}\sin\;(\omega t)$ and $r_{2}(t)=r_{2}+a_{2}\sin\;(\omega t+\phi )$, where *ω* is the frequency of the underlying oscillation and *ϕ* is the phase lag (Figs. 2a and b). The CC for the spike trains derived from the above-mentioned two processes can be obtained analytically as $c_{12}(t)=r_{1}r_{2}+\frac{a_{1}a_{2}}{2}\cos\;(\omega t+\phi )$, thus reproducing the slow undulation. Note that this is a theoretical result obtained for spike trains of infinite length. CCs constructed from a finite number of spikes exhibit noisy fluctuations. Nevertheless, such fluctuations can be minimized if we can obtain spike trains of sufficiently long duration.

The slow undulation appearing in biological CCs generally exhibits damping because the underlying oscillation is not purely harmonic. For the cases in which frequencies are distributed normally around *ω* with variance $1/\delta^{2}$, the CC exhibits damping, as represented by the Gabor function, $c_{12}(t)=r_{1}r_{2}+\frac{a_{1}a_{2}}{2}e^{-t^{2}/2\delta^{2}}\cos\;(\omega t+\phi )$. While many biological CCs show a peak at the origin, there are cases in which the peak is shifted from the origin. Thus two neurons exhibiting a peak shifted from the origin of the CC may have been receiving brainwave oscillations with a fixed latency.

#### Models that produce a cusp in a CC

Even if the undulation of a biological CC does not seem simply harmonic, any smooth shape can be represented as a sum of many oscillatory components. Accordingly, undulations of nontrivial shape may also be explained such that the neurons may have been influenced by many brainwave components. However, the cusp we found in many biological CCs cannot be explained as a simple combination of a finite number of smooth brainwave components. Instead, the original processes should be “mean-square differentiable” (30). We have explored mathematical models that can produce a cusp at the origin of the CC. For simplicity’s sake, we assume that a pair of neurons generate spikes according to identical time-dependent rates. While their rate processes may contain independent parts in practice, they should have common inputs of this kind.

In the first model, the firing rates of two neurons follow the Ornstein–Uhlenbeck process (49), as given by the stochastic differential equation, $dr(t)/dt=-2(r(t)-\mu )/\tau_{r}+2(\sigma /\sqrt{\tau_{r}})\xi (t)$, where *μ*, *σ*, and $\tau_{r}$ are the mean, standard deviation, and the time scale of the fluctuation. ξ(t) is Gaussian white noise characterized by ensemble averages ⟨ξ(t)⟩=0 and $\left\langle \xi (t)\xi (t^{\prime })\rangle =\delta (t-t^{\prime }\right)$. The rate is continuous but is nondifferentiable everywhere (Fig. 2c). In this case, the CC of two neurons generating spikes according to the identical rate processes r(t) can be obtained analytically as (Fig. 2c) $c_{ij}(t)=\mu^{2}+\sigma^{2}e^{-2|t|/\tau_{r}}$, exhibiting a cusp at the origin (51–53). These two neurons are assumed to have no synaptic connections because they are generating spikes independently according to instantaneous rates.

The cusp in the CC appears at the origin if two neurons are driven coherently, but the location of the cusp can be shifted from the origin if two neurons receive fluctuations with some time lag. However, we have rarely seen experimental data in which the cusp is shifted from the origin, although we have seen some cases in which the peak of the oscillatory undulation is shifted from the origin.

In the second model, the firing rates of two neurons follow a switching rate process in which the rate changes discontinuously between high and low values r(t)=μ+σ or μ−σ according to the “Markov switching process” or “random telegraph process” with a mean time interval $\tau_{r}$ (53–57) (Fig. 2d). In this case, the inter-transition interval *t* is distributed exponentially as $p(t)=1/\tau_{r}e^{-t/\tau_{r}}$. In this case, the CC of two neurons generating spikes becomes $c_{ij}(t)=\mu^{2}+\sigma^{2}e^{-2|t|/\tau_{r}}$, which is identical to the CC of the Ornstein–Uhlenbeck process.

In the Ornstein–Uhlenbeck process, the time-dependent rate is nondifferentiable everywhere. In the Markov transition process, the rate changes discontinuously at a finite rate. It is noteworthy that such different processes may give rise to an identical cusp in CCs. A common characteristic of the underlying rate processes for producing a cusp in the CC is that the firing rates of two neurons undergo coherent nondifferentiable rates.

The original rate process cannot be identified uniquely from a CC, because a CC is obtained by averaging over a time series. Even though we cannot visualize the original rate processes from recorded spike trains, we can obtain a sharp profile of the CC from sufficiently long spike sequences because the number of spike counts in each bin of the CC increases with the length of the spike trains (Supplementary Material A).

### Methods of estimating monosynaptic connectivity

#### GLMCC

Because Classical CC analysis has made many false inferences due to large undulations in CCs, modern analysis methods attempted to eliminate the influence of the undulations. For instance, the GLMCC method (28) introduced a function a(t) to absorb the large undulation in an original CC and tried to fit the function $\lambda (t)=\exp\;\left(a(t)+J_{ij}f(t)+J_{\;ji}f(-t)\right)$ to $c_{ij}(t)$ obtained for a given pair of neurons. Here $J_{ij}$ represents a discrete impact due to possible monosynaptic connectivity from the *j*th neuron to the *i*th neuron, and f(t) is the time profile of the synaptic interaction, modeled as $f(t)=\exp\;(-\frac{t-d}{\tau_{s}})$ for t>d and f(t)=0 otherwise, where $\tau_{s}$ is a typical timescale of synaptic impact and *d* is the transmission delay. For making a fitting function a(t) represent a smooth undulation in the CC, a large gradient of a(t) is penalized with the prior distribution $\exp\;\left[-\frac{1}{\gamma }\int_{-W}^{W}(\frac{da}{dt}{)}^{2}\;dt\right]$, where *W* is the time window of a CC and *γ* is a hyperparameter representing the flatness of a(t); we selected here as W=50 ms and $\gamma =5\times 10^{-4}$ [ms^−1^]. The set of parameters $\left\{J_{12},J_{21},a(t)\right\}$ was determined by maximizing the posterior distribution given the spike data $\left\{t_{k}\right\}$ in the CCs (28) or its logarithm: $\sum_{k}\log\;\lambda (t_{k})-\int_{-W}^{W}\lambda (t)\;dt-\frac{1}{\gamma }\int_{-W}^{W}(\frac{da}{dt}{)}^{2}\;dt$.

#### ShinGLMCC

While the original GLMCC algorithm solved the problem of undulation by fitting a smooth function evenly to the original CC, it cannot reproduce a cusp at the origin. Therefore, we have revised it into ShinGLMCC by allowing the function a(t) to bend at the origin. Specifically, we separate a single integral in the prior distribution into two pieces of half-lines and changed the first-order derivative to the second-order derivative, $\exp\;[-\frac{\beta }{2}(\int_{-W}^{-0}(\frac{d^{2}a}{dt^{2}}{)}^{2}\;dt+$  $\int_{+0}^{W}(\frac{d^{2}a}{dt^{2}}{)}^{2}\;dt)]$, with the constraint that the value a(t) is continuous at the origin, a(−0)=a(+0). This means that a(t) is requested to be smooth in each half region, except at the origin. Because the order of derivative changed, we have selected a new penalizing parameter of different dimensionality, $\beta =10^{6}$ [ms^3^]. We have devised a numerical code enabling fast estimation of ShinGLMCC.

While the new fitting function a(t) optimized by the new ShinGLMCC may look similar to the one optimized by the original GLMCC, they can be different particularly near the origin, deriving different decisions for detecting connections in some cases as demonstrated in Figs. 1h–j.

#### Likelihood ratio test

In the original GLMCC, the presence of connectivity was determined by thresholding the estimated connectivity parameter $\left|J_{ij}\right|$ depending on the recording time, and firing rates of pre and post-synaptic neurons (28). However, this thresholding causes an asymmetry in detectability between excitatory and inhibitory connections. Accordingly, GLMCC was revised by introducing the likelihood ratio test such that the connection is determined based on the likelihood ratio between the presence and absence of the connectivity, $D=\log\;L(J_{ij}={\hat{J}}_{ij})-\log\;L(J_{ij}=0)$, where ${\hat{J}}_{ij}$ is the estimated connection parameter and *L* is the model likelihood (29). Based on Wilks’ theorem (65), we may reject a null hypothesis that a connection is absent (or determine that the connection is likely to present) if $2D>z_{\alpha }$, where $z_{\alpha }$ is the threshold of $\chi^{2}$ distribution of a significance level *α*.

The ShinGLMCC algorithm also adopts the likelihood ratio test with $\alpha =10^{-4}$. We also searched for the timescale of synaptic impact $\tau_{s}$ out of 1, 2, 3, and 4 ms, and the transmission delay *d* out of 1, 2, and 3 ms, based on the likelihood ratio.

### Evaluating analysis methods

The performance of estimation methods was evaluated using the data obtained from simulations of a large-scale network of neurons. For this purpose, we have counted the number of estimation failures such as FPs and FNs for excitatory and inhibitory categories (for the excitatory category, we considered only the case where the excitatory conductance is greater than 0.01). These failures are quantified by FPR and FNR the smaller the better. As a total score of the goodness of estimation, we computed the Matthews correlation coefficient (MCC) (66), the larger, the better. We have taken the macro-average MCC that gives equal importance to excitatory and inhibitory categories, $\mathrm{MCC}=({\mathrm{MCC}}_{e}+{\mathrm{MCC}}_{i})/2$.

### Simulating a large-scale network of neurons

To evaluate estimation methods such as Classical CC, GLMCC, and ShinGLMCC in their accuracy in detecting connectivity, we have generated spike trains from 1,000 model neurons interacting with fixed synaptic connectivity. While the model simulation is similar to that of Ref. (29), we carried out an independent simulation with the following conditions.

As a spiking neuron model, we adopted the Multitimescale Adaptive Threshold (MAT) model (67, 68). In this model, “membrane potential” $v_{m}$ obeys a simple leaky integration of input signal without resetting: $\frac{dv_{m}}{dt}=-\frac{\left(v_{m}-V_{L}\right)}{\tau_{m}}-[g_{e}(v_{m}-V_{E})+g_{i}(v_{m}-V_{I})]-\frac{RI_{\mathrm{bg}}}{\tau_{m}}$, where $g_{e}$ and $g_{i}$ represent the excitatory and inhibitory conductances, respectively, whereas $RI_{\mathrm{bg}}$ represents the background inputs coming from outside the population. The conductance evolves with $\frac{dg_{X}}{dt}=-\frac{g_{X}}{\tau_{s,X}}+\sum_{j}\sum_{k}G_{\;j}\delta (t-t_{\;jk}-d_{\;j})$ where *X* stands for excitatory *e* or inhibitory *i*  $\tau_{s,X}$ is the decay constant, $t_{\;jk}$ is the *k*th spike time of *j*th neuron, $d_{\;j}$ is a synaptic delay and $G_{\;j}$ is the synaptic weight from *j*th neuron. δ(t) is the Dirac delta function.

In this model, the threshold for generating spikes θ(t) is lifted when generating a spike and subsequently decays with two timescales as $\theta (t)=\sum_{j}H(t-t_{j})+\omega$, $H(t)=\sum_{k=1,2}\alpha_{k}\exp\;(-t/\tau_{k})$, where $t_{j}$ is the *j*th spike time of a neuron, *ω* is the resting value of the threshold, $\tau_{k}$ is the *k*th time constant, and $\alpha_{k}$ is the weight of the *k*th component (k=1,2). The shorter timescale $\tau_{1}=10$ ms represents the membrane time constant, and the longer timescale $\tau_{2}=200$ ms represents the adaptation of neuronal spiking. Here we have adopted the parameters of the MAT model such that excitatory neurons are distributed between the regular spiking (RS) and intrinsic bursting (IB) neurons and inhibitory neurons are the fast-spiking (FS) neurons, as represented in Fig. 5 of Ref. (67). The parameter values chosen for the present simulation are summarized in Table 1.

Among 1,000 neurons, 800 excitatory neurons innervate to 12.5% of other neurons with the conductances that are log-normally distributed (63, 69, 70), whereas 200 inhibitory neurons innervate randomly to 25% of other neurons with the conductances that are normally distributed.

The excitatory and inhibitory synaptic connections were sampled from the respective distributions. The excitatory conductances $\left\{G_{ij}^{E}\right\}$ were sampled from a log-normal distribution with the mean −5.543 and SD 1.30 of the natural logarithm of the conductances (63, 69). The inhibitory conductances $\left\{G_{ij}^{I}\right\}$ were sampled from the normal distribution with the mean 0.0217 and SD of 0.00171. If the value sampled from the normal distribution is negative, it is resampled from the same distribution. The entire connection matrix $\left\{G_{ij}\right\}$ is multiplied by the strength constant *A*, which we changed from 0.6 to 1.8 to see if the network exhibits nonstationary burst firing.

The synaptic delays from excitatory neurons are distributed uniformly between 3 and 5 ms, and those from inhibitory neurons are distributed uniformly between 2 and 4 ms.

We added a background current to represent random bombardments from excitatory and inhibitory neurons of the outside population (71), $RI_{\mathrm{bg}}=g_{e}^{\mathrm{bg}}(v_{m}-V_{E})+g_{i}^{\mathrm{bg}}(t)(v_{m}-V_{I})$. The dynamics of the conductances can be approximated as a stationary fluctuating process represented by the Ornstein–Uhlenbeck process, $\frac{dg_{X}^{\mathrm{bg}}}{dt}=-\frac{g_{X}^{\mathrm{bg}}-g_{X,0}^{\mathrm{bg}}}{\tau_{s,X}^{\mathrm{bg}}}+\sqrt{\frac{2{\sigma_{X}^{\mathrm{bg}}}^{2}}{\tau_{s,X}^{\mathrm{bg}}}}\xi (t)$, where $g_{X}$ stands for $g_{e}$ or $g_{i}$, and ξ(t) is the white Gaussian noise satisfying ⟨ξ(t)⟩=0 and ⟨ξ(t)ξ(s)⟩=  δ(t−s). The parameter values for the background inputs are summarized in Table 2.

**Table 2.** Parameters for background inputs.

$\tau_{s,e}^{\mathrm{bg}}$ , $\tau_{s,i}^{\mathrm{bg}}$ [ms]	2.7, 10.5
$g_{e,0}^{\mathrm{bg}}$ , $g_{i,0}^{\mathrm{bg}}$	1.85, 4.83
$\sigma_{e}^{\mathrm{bg}}$ , $\sigma_{i}^{\mathrm{bg}}$	0.245, 0.400

The numerical simulation of a given network was carried out for a time interval of 3,600 s with a time step of 0.0001 s.

### Biological data

We have analyzed publicly sourced data of spike trains recorded in parallel from the brain of a freely moving rat using a silicon probe called Neuropixels (2, 47). They recorded multiple neurons from the visual cortex, hippocampus, and thalamus in an awake mouse. We analyzed spike trains of 242 units according to their evaluation as being well isolated. We also analyzed other data of Ref. (2, 47) recorded from the frontal cortex including motor areas and the posterior cortex including visual areas (Supplementary Material D).

## Supplementary Material

pgae261_Supplementary_Data

## Data Availability

We have developed the ShinGLMCC algorithm using Python. We also revised the GLMCC algorithm so that the computation can be carried out much faster than the original one developed in Ref. (28, 29). Additionally, we developed the network simulation code in Python. All codes are available in [https://github.com/yasuhirotsubo/neuroscience] such that connection matrices estimated by ShinGLMCC and GLMCC can be computed for a given set of spike trains.

## Preprints

A preprint of this article is published at [https://www.biorxiv.org/content/10.1101/2023.06.12.544700v1.abstract].
