# Supplementary material for: Nondifferentiable activity in the brain
Source: PNAS Nexus. 2024 Jul 1;3(7):pgae261. doi: 10.1093/pnasnexus/pgae261 (PMC11238849; doi:10.1093/pnasnexus/pgae261)
Supplement: pgae261_Supplementary_Data [file pgae261_supplementary_data.pdf]

Supporting Information for

Non-differentiable activity in the brain

Yasuhiro Tsubo and Shigeru Shinomoto

Yasuhiro Tsubo
Email: tsubo@fc.ritsumei.ac.jp

Shigeru Shinomoto
Email: shigerushinomoto@gmail.com

This PDF file includes:

- Supporting text
- Figures S1 to S9
- SI References

Supporting Information Text

A. Spike count fluctuations in cross-correlograms.

Interdependence of neuronal firings may be detected in their cross-correlogram, which can be constructed by superposing the spike times of one train measured relative to every spike of another train, as depicted in Fig. 1a in the main text. However, the cross-correlogram obtained from a finite recording duration is inevitably accompanied by spike count fluctuations. Here we estimate the degree of spike count fluctuations that occur in each time bin Δ for two spike trains of mean firing rates r_1 and r_2, recorded for a duration T.

If the two spike trains are independent, their cross-correlogram is expected to be flat, and the expected number of spikes counted in each bin is $\lambda = r_1 \Delta r_2 T$. Because the spike count in each bin is a random process, it follows the Poisson distribution, where the variation is identical to the mean. Accordingly, the degree of spike count fluctuations as represented by the standard deviation (SD) divided by the mean is given by Reference (1)

$$\delta\lambda/\lambda = 1/\sqrt{r_1 \Delta r_2 T}.$$

Since this trend holds even when there is a systematic change in the cross-correlation, the systematic part becomes visible if the recording duration T is increased.

Here we take up model cases in which neurons are modulated by smooth oscillations and non-differential fluctuations as exemplified in Figs. 2a and 2c, and show how their cross-correlograms change with the recording duration. In Fig. S1, we observe that the systematic parts in the cross-correlations become more visible as the recording duration is increased from 1, 10, to 100 hours. The fluctuations in the middle figures are larger than those of Fig. 2a and 2c because the bin size ($\Delta = 1$ ms) is smaller than that of Fig. 2a or 2c ($\Delta = 10$ ms). In these cases, the cross-correlations of the smooth undulation and a non-smooth cusp that should be obtained at the limit of infinite recording duration are obtained analytically (METHODS), and we have depicted them in the red lines.

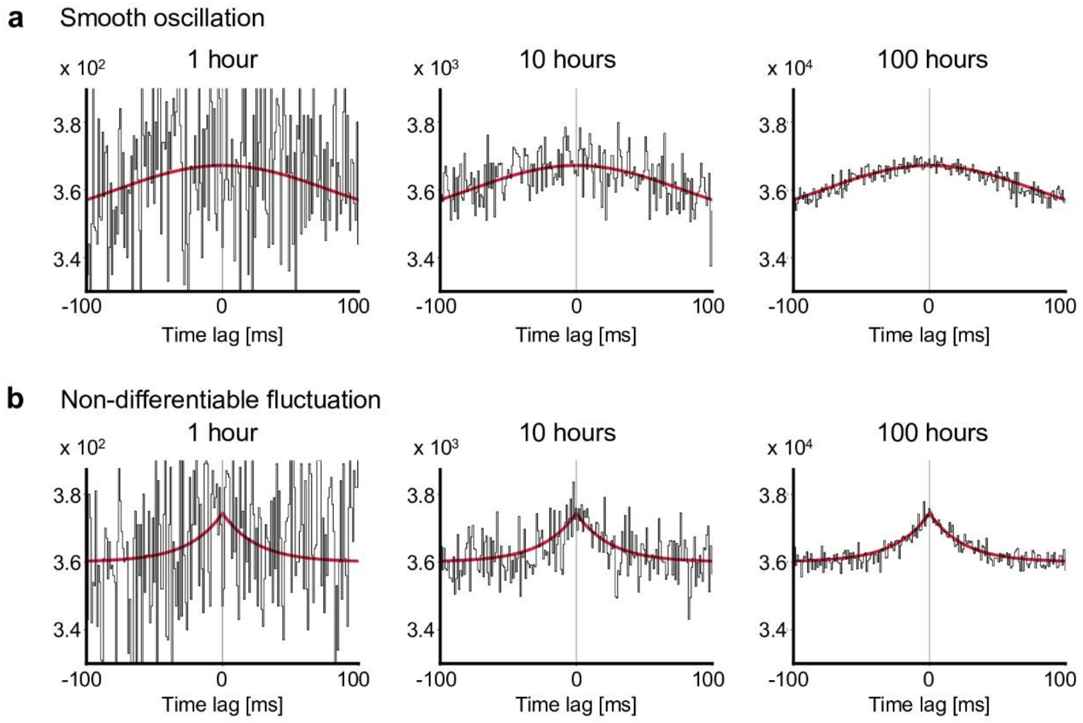

Fig. S1. Close-up views of cross-correlation histograms obtained with different recording durations. Cross correlograms of spike trains modulated with (**a**) smooth oscillations and (**b**) non-differentiable fluctuations as demonstrated in Figs. 2**a** and 2**c**. Observation duration T was varied from 1, 10, to 100 hours. Red lines are analytically obtained CCs for individual models, as was obtained in METHODS.

B Autocorrelation histograms obtained with spike time dithering.

To examine whether the central peak of the autocorrelation in Fig. 3b is a non-smooth cusp or it is just a smooth hump, we apply jittering or dithering to a summed spike train so that individual spike times are randomly shifted around the original times (References 2-4), and see how the peak of the autocorrelation histogram collapses according to the dithering. The SDs of the dithering times are 5, 10, and 50 ms. It is observed in Fig. S2 that an apparently non-smooth cusp in the original autocorrelation function collapsed into a smooth hump, even with the short dithering of 5 or 10 ms. This suggests that there is a sharp coherent activity in the brain on the order of a few milliseconds.

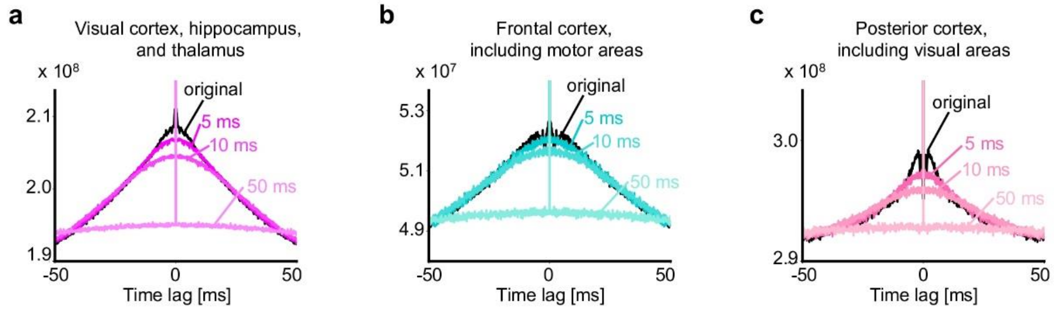

Fig. S2. Close-up images of autocorrelation histograms of dithered spike times. Autocorrelation histograms were computed for dithered spike trains in which individual spike times were randomly displaced with SDs of 5, 10, and 50 ms. The autocorrelation histograms are plotted for an interval of [-50, 50] ms with a 0.1 ms bin. **a** Visual cortex, hippocampus, and thalamus (close-up view of Fig. 3b); **b** Frontal cortex including motor areas; and **c** Posterior cortex including visual areas.

C Fitting differentially discontinuous and continuous functions to autocorrelation histograms.

To provide a way to determine non-differentiable activity from discrete time data, which may also be associated with spike sorting errors, we have compared a piecewise differentially discontinuous function with a differentially continuous function for their likelihood or the goodness of fit to the autocorrelation histogram of the summed spike train (with the delta peak that is due to self-spike counts is removed). We have chosen the piecewise differentially discontinuous function as in ShinGLMCC with a coefficient $\beta = 10^8$, while the differentially continuous function as having no break in the prior distribution of the second order derivative. As a result, we have confirmed that the central peak of the autocorrelation histogram may be better represented by a piecewise differentially discontinuous function than by a differentially continuous function.

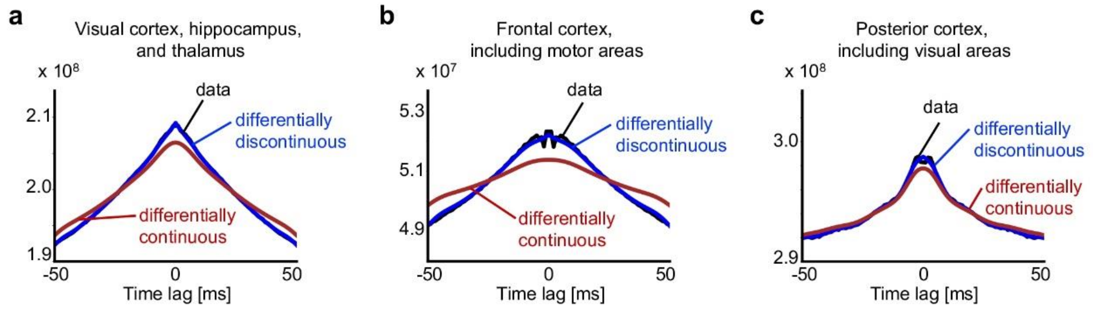

Fig. S3. Fitting differentially discontinuous and continuous functions to autocorrelation histograms. Piecewise differentially discontinuous functions and differentially continuous functions are fitted to autocorrelation histograms of summed spike trains. The data sets are the same as those in Fig. S2. The likelihood of the differentially discontinuous function is higher than that of the differentially continuous function by 24724 and 28964, and 1917 for a, b, and c, respectively.

D Analysis of other data sets recorded in different brain regions.

Here we analyze two sets of spike trains in References (5, 6) recorded from brain regions that are different from the data analyzed in the main text (the visual cortex, hippocampus, and thalamus). The recorded regions were (A) the frontal cortex including motor areas (Figs. S4 and S5) and (B) the posterior cortex including visual areas (Figs. S6 and S7).

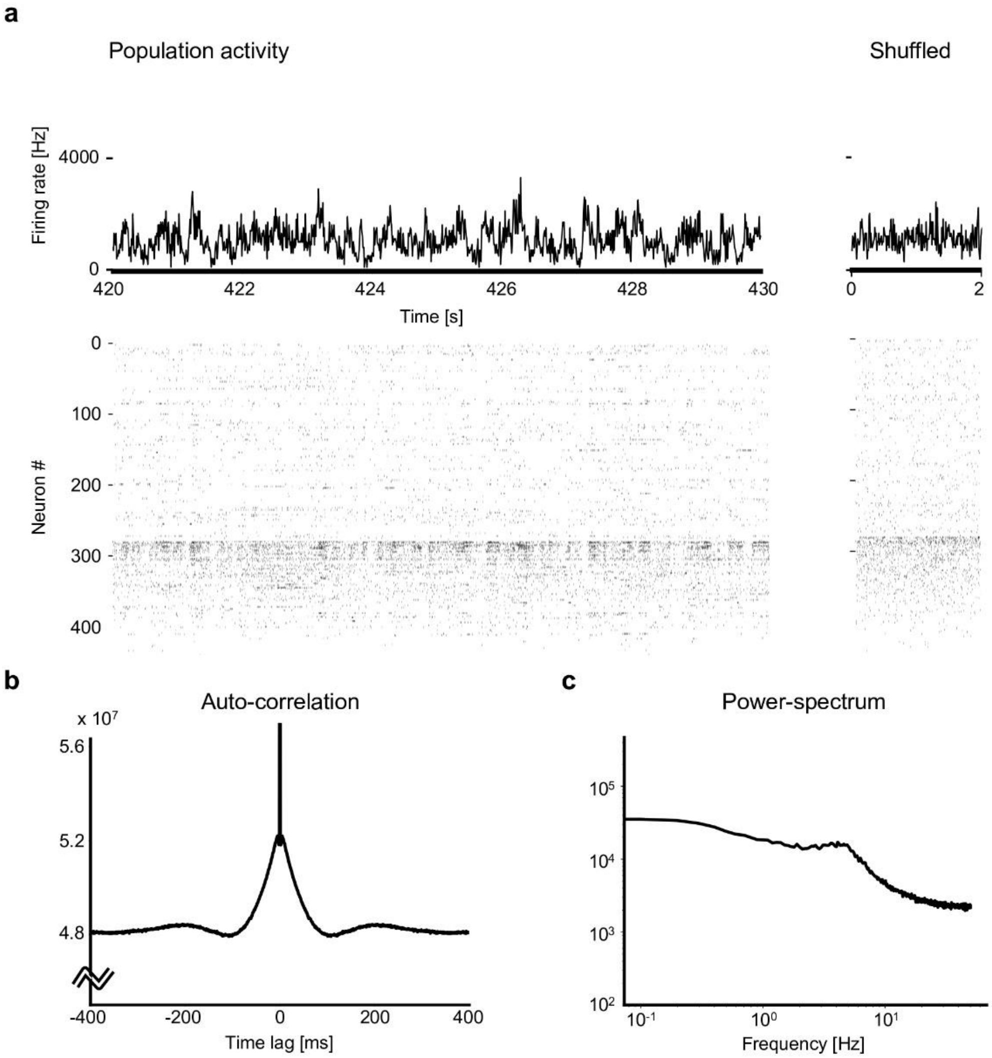

Fig. S4. The population activity of the frontal region including motor areas. a (Left-top) A time histogram for 10 s, with a time bin of 10 ms. (Left-bottom) Spike rasters. (Right-top) A time histogram of a shuffled spike train for 2 s. (Right-bottom) Rasters of shuffled spike trains. **b** and **c** An autocorrelation histogram (1ms bin) and a power spectrum of the summed spike train.

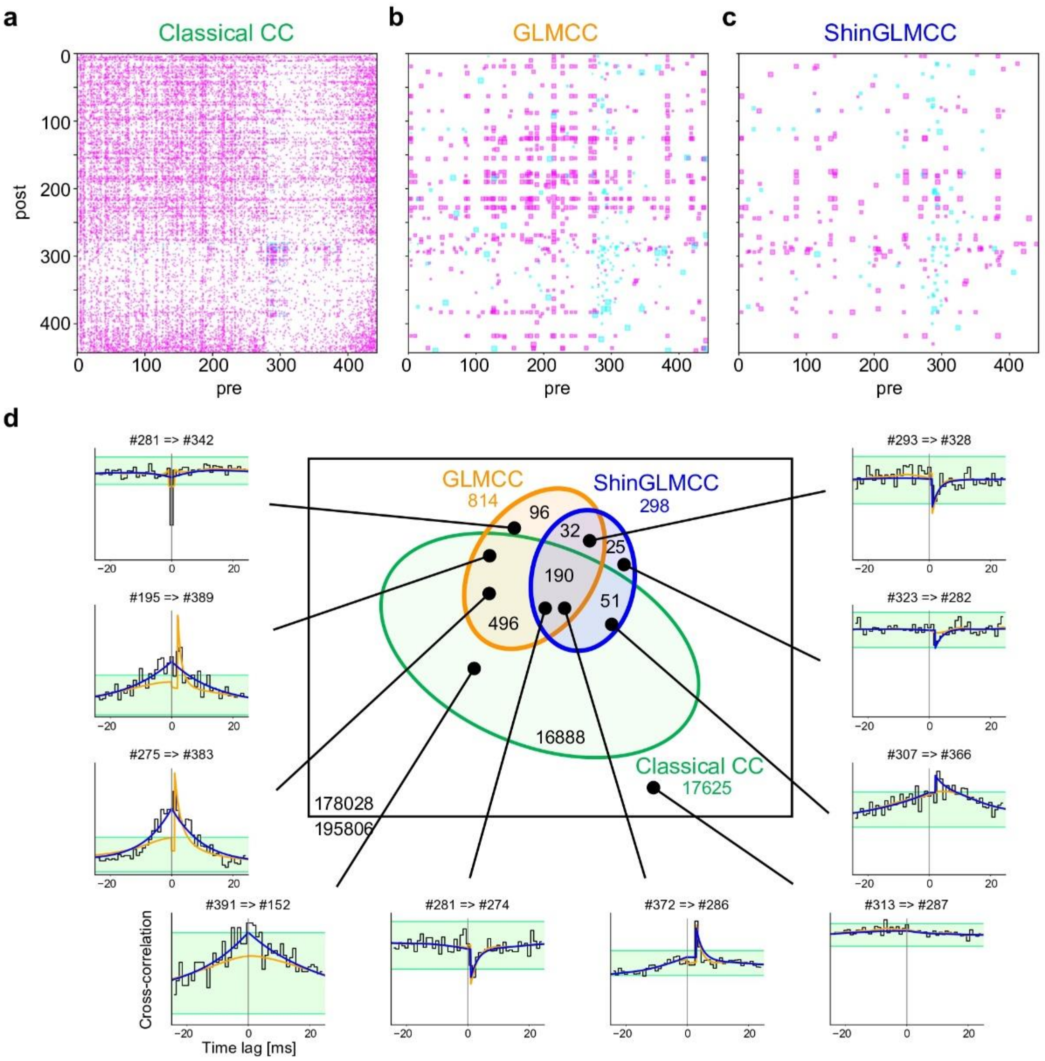

Fig. S5. Comparison of analysis methods for the frontal region including motor areas. a-c Connection matrices obtained for biological data using the Classical CC method, the GLMCC method, and the newly developed ShinGLMCC method, respectively. Magenta and cyan squares in each matrix represent excitatory and inhibitory connections, respectively estimated by each analysis method. **d** Venn diagram representing the relationships between determined connectivities. Sample CCs are displayed along the periphery.

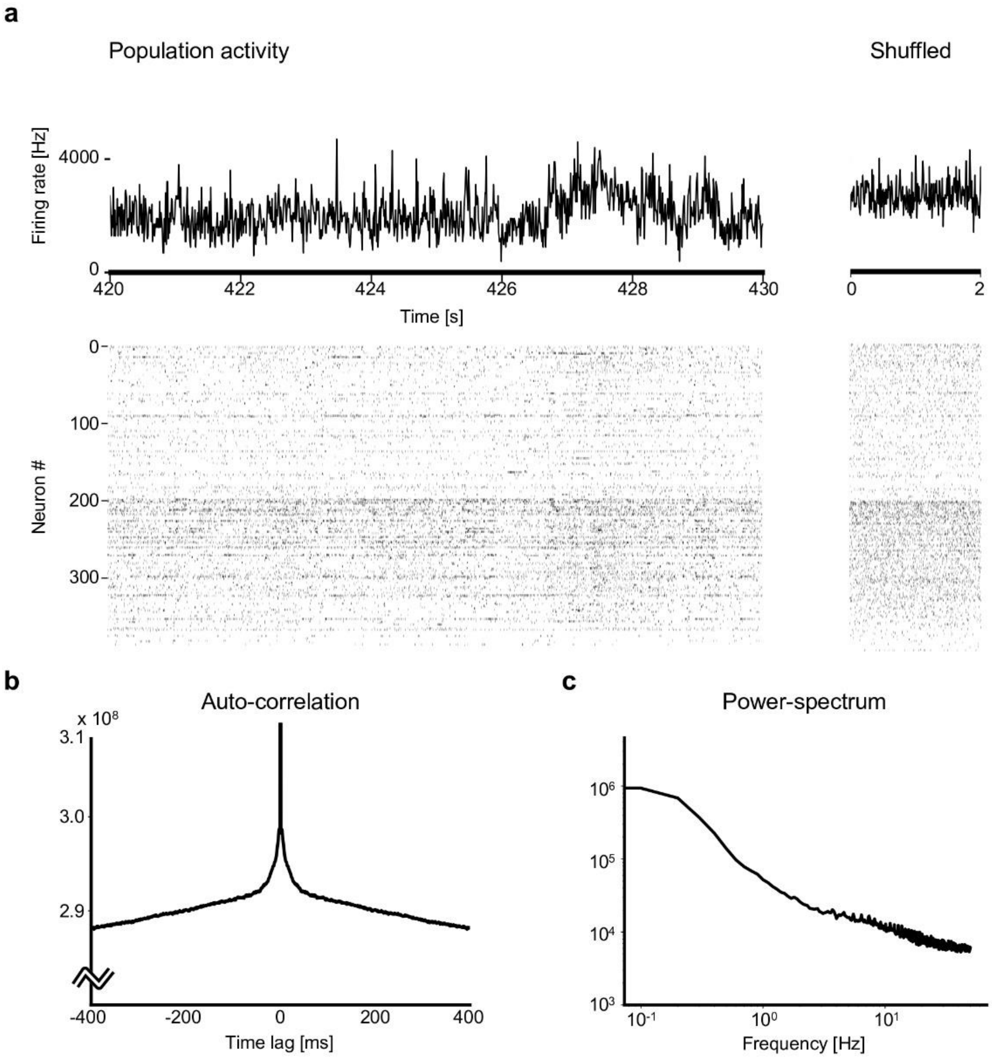

Fig. S6. The population activity of the posterior region including visual areas. a (Left-top) A time histogram for 10 s, with a time bin of 10 ms. (Left-bottom) Spike rasters. (Right-top) A time histogram of a shuffled spike train for 2 s. (Right-bottom) Rasters of shuffled spike trains. **b** and **c** An autocorrelation histogram (1ms bin) and a power spectrum of the summed spike train.

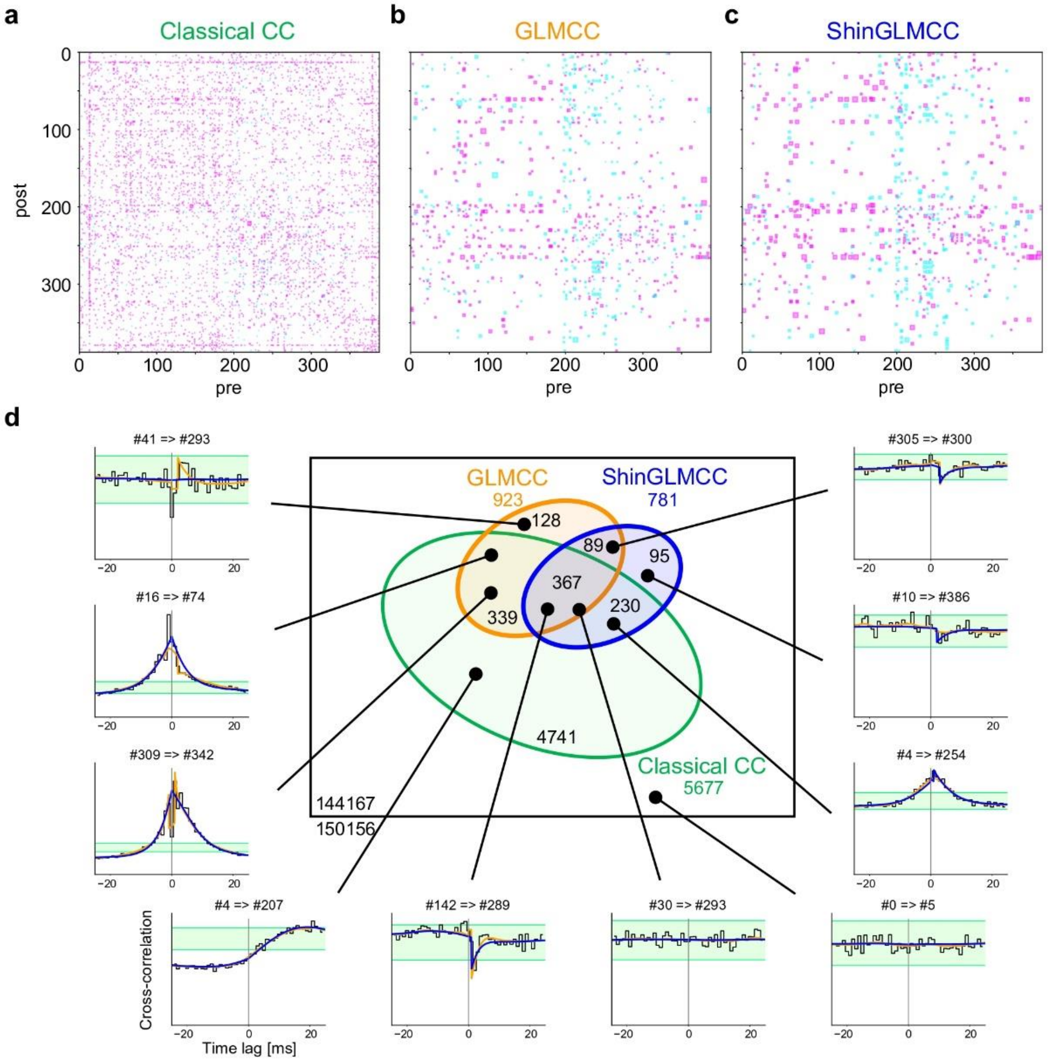

Fig. S7. Comparison of analysis methods for the posterior region including visual areas. a-c Connection matrices obtained for biological data using the Classical CC method, the GLMCC method, and the newly developed ShinGLMCC method, respectively. Magenta and cyan squares in each matrix represent excitatory and inhibitory connections, respectively estimated by each analysis method. **d** Venn diagram representing the relationships between determined connectivities. Sample CCs are displayed along the periphery.

E . Comparison of ShinGLMCC and GLMCC in their goodness of fit to the cross-correlograms.

To see the difference in the local dynamics of neuron pairs, we have compared ShinGLMCC and GLMCC for their goodness of fit to individual cross-correlograms. Figure S8 shows matrices indicating the neuron pairs for which ShinGLMCC fits the cross-correlogram better (the likelihood is higher) than GLMCC. Here, neurons are sorted as linearly aligned along the Neuropixel. There are significant differences in the cusp cross-correlogram dominance between different brain regions.

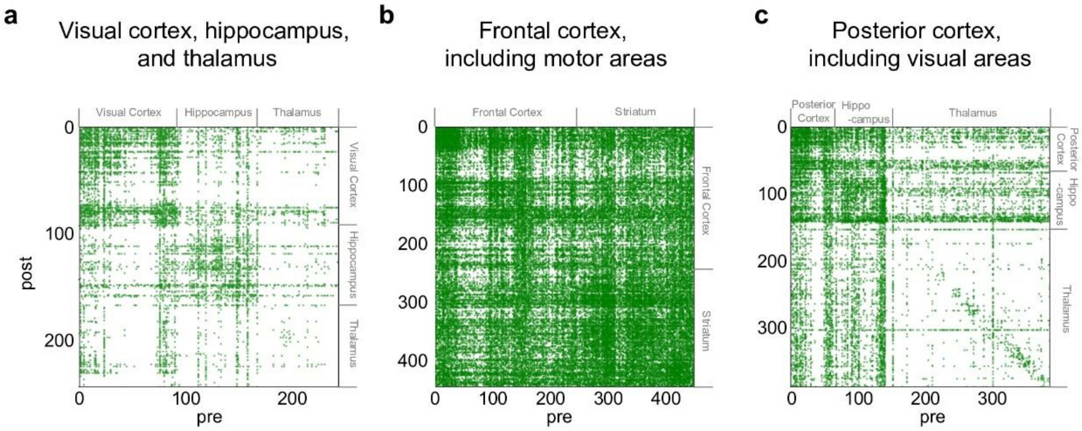

Fig, S8. Matrices indicating the neuron pairs for which ShinGLMCC better fits the cross-correlograms. a Visual cortex, hippocampus, and thalamus (close-up view of Fig. 3b); **b** Frontal cortex including motor areas; and **c** Posterior cortex including visual areas.

F Consistency of identified connections.

We have tested how the excitatory or inhibitory characteristics of identified connections are consistent for individual neurons, by computing the excitatory-inhibitory (E-I) dominance index proposed in Reference (7), defined as

$$d_{\mathrm{ei}} = (n_{\mathrm{e}} - n_{\mathrm{i}})/(n_{\mathrm{e}} + n_{\mathrm{i}})$$

where n_{e} and n_{i} are the numbers of identified excitatory and inhibitory connections projecting from each neuron, respectively. We have shown the distributions of the E-I dominance index in Fig. S9. The Classical CC, GLMCC, and ShinGLMCC gave connections to 241, 189, and 142 neurons out of a total of 242 neurons. The average absolute values of E-I dominance $|d_{\mathrm{ei}}|$ were 0.78, 0.81, and 0.86, and the fractions of neurons expressing perfect consistency ($d_{\mathrm{ei}} = 1$ or -1) were 0.28, 0.69, and 0.75, respectively, suggesting that ShinGLMCC may have provided a reliable inference.

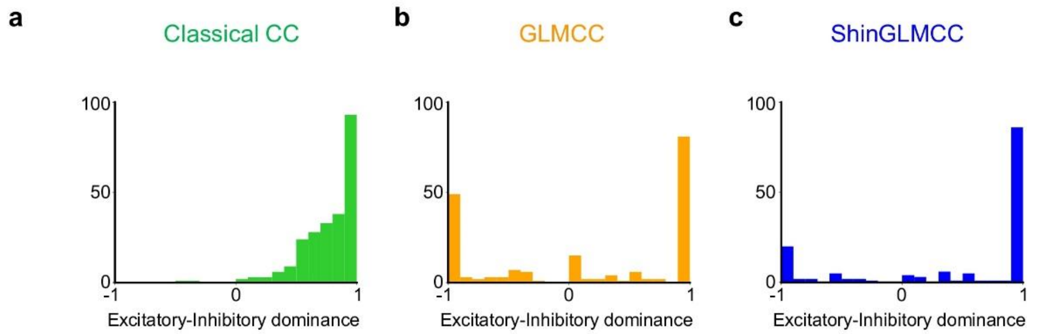

Fig. S9. Comparison of analysis methods. Distributions of the excitatory--inhibitory (E-I) dominance indices $d_{\mathrm{ei}} = (n_{\mathrm{e}} - n_{\mathrm{i}})/(n_{\mathrm{e}} + n_{\mathrm{i}})$ computed for (**a**) Classical CC, (**b**) GLMCC, and (**c**) ShinGLMCC.

SI References

1. Ad MHJ Aertsen and George L Gerstein. Evaluation of neuronal connectivity: sensitivity of cross-correlation. *Brain Res*., 340(2):341–354, 1985.
2. George L Gerstein and Ad M Aertsen. Representation of cooperative firing activity among simultaneously recorded neurons. *Journal of Neurophysiology*, 54(6):1513–1528, 1985.
3. Antonio Pazienti, Pedro E Maldonado, Markus Diesmann, and Sonja Grün. Effectiveness of systematic spike dithering depends on the precision of cortical synchronization. *Brain research*, 1225:39–46, 2008.
4. Sonja Grün. Data-driven significance estimation for precise spike correlation. *J. Neurophysiol*., 101(3):1126–1140, 2009.
5. James J Jun, Nicholas A Steinmetz, Joshua H Siegle, Daniel J Denman, Marius Bauza, Brian Barbarits, Albert K Lee, Costas A Anastassiou, Alexandru Andrei, Cagatay Aydın, et al. Fully integrated silicon probes for high-density recording of neural activity. *Nature*, 551(7679):232, 2017.
6. Nick Steinmetz, Matteo Carandini, and Kenneth D. Harris., "Single Phase3" and "Dual Phase3" *Neuropixels Datasets*. "https://figshare.com/articles/_Single_Phase3_Neuropixels_Dataset/7666892", doi = "10.6084/m9.figshare.7666892.v2 (2019).
7. Ryota Kobayashi, Shuhei Kurita, Anno Kurth, Katsunori Kitano, Kenji Mizuseki, Markus Diesmann, Barry J Richmond, and Shigeru Shinomoto. Reconstructing neuronal circuitry from parallel spike trains. *Nature communications*, 10(1):1–13, 2019.
